# Psychological Well-Being and Type 2 Diabetes

**DOI:** 10.19080/crdoj.2017.04.555641

**Published:** 2017-10-30

**Authors:** Christina N Massey, Emily H Feig, Laura Duque-Serrano, Jeff C Huffman

**Affiliations:** Department of Psychiatry, Massachusetts General Hospital, Harvard Medical School, USA

**Keywords:** Diabetes, Positive psychology, Well-being, Positive affect, Positive psychology intervention, Optimism

## Abstract

Positive psychological characteristics such as optimism, positive affect, gratitude, and related constructs may play an important role in health. In patients with type 2 diabetes (T2D), positive psychological constructs have been associated with superior medical outcomes, including better glucose control and lower mortality rates. The beneficial effects of positive psychological states in T2D are most likely mediated through health behaviors such as increased physical activity and adherence to a healthier diet. Furthermore, numerous studies with non-diabetic populations have shown that performing various positive psychological exercises (e.g., writing gratitude letters, performing acts of kindness) have led to greater well-being. Compared to other available treatments, these activities are simple and involve constructs that have been associated with superior adherence and diabetes-related outcomes. However, there has been minimal research on the use of positive psychological interventions in T2D, though small studies of related interventions have been linked to improvements in positive affect and, in some cases, greater health behavior adherence and lower blood sugar. Continued work is needed to ascertain whether positive psychology interventions can truly impact functioning, blood sugar, and overall health in this key population.

Psychological distress and negative affective disorders are common in patients with type 2 diabetes (T2D). A substantial proportion of patients with T2D have clinical depression [1] and even those who do not meet full diagnostic criteria for a depressive disorder have substantial distress that can impede self-care, functioning across multiple domains (e.g., occupational, personal), and quality of life [2]. Psychological states may also significantly impact health behavior and clinical outcomes in patients with T2D [3]. Negative psychological syndromes such as depression and anxiety have been consistently associated with poor outcomes in patients with T2D [4–6]. For example, depression is associated with impaired glucose control [7], functional disability [8], end-organ complications [7], and mortality [7,9,10], and distress itself is associated with lower levels of treatment adherence [2].

On the other hand, positive psychological characteristics-optimism, positive affect, gratitude, and related constructs-may also play an important role in medical outcomes. These positive psychological constructs are not simply the flip-side of depression [11,12], e.g., it is possible that a depressed individual may be optimistic about the future whereas a non-depressed individual may conversely have low levels of optimism. Prior work has found that these constructs have been linked to superior health outcomes including healthier diet, increased physical activity, and lower rates of mortality across various medical conditions [13–15]. Furthermore, the connections between positive psychological constructs and health have been independent of sociodemographic factors, medical characteristics, and the adverse effects of depression and anxiety [13,15]. Nonetheless, there has been less focus on the promotion of positive psychological well-being in T2D individuals.

Specifically related to T2D, positive psychological constructs have been associated with numerous beneficial outcomes [3,16–18]. For example, in a large epidemiologic study, measures of psychological well-being, including emotional vitality and life satisfaction, were prospectively linked with the prevention of T2D [19]. Among patients who have developed T2D, positive psychological attributes are related to superior outcomes. For instance, overall well-being is correlated with better glucose control [20]. Likewise, resilience has been associated with lower levels of hemoglobin A1c, and such resilience has been shown to buffer the effects of psychological distress on blood sugar [21]. Finally, positive affect has been prospectively and independently linked to lower mortality among those with T2D [22].

Though the relationship of positive psychological constructs and health outcomes is not fully understood, the beneficial effects of positive psychological states are most likely mediated through health behaviors. Positive states in some studies have been directly associated with favorable effects on physiology in T2D (e.g., reduced sympathetic hyperactivity, decreased levels of proinflammatory biomarkers, and decreased hypothalamic-pituitary-adrenal axis hyperactivity) [3,16–18]. However, most evidence that links positive states to superior outcomes does so via increased adherence to health behaviors [16–18]. For example, in T2D patients, positive affect and optimism have been prospectively linked to greater physical activity, healthier diet, and reduced smoking, even after controlling for baseline behavior and relevant covariates [22]. Such improvements in health behavior may be caused by easier initiation of physical activity, greater confidence in meeting diet and activity goals, and more vitality/energy to engage in self-management when experiencing positive mood [23].

An important question is whether positive psychological well-being is inherent or whether it can be modified. There is by now a substantial literature on so-called positive psychology (PP) interventions and their efficacy in improving psychological well-being. PP interventions use exercises (e.g., gratitude letters, acts of kindness, personal strengths), completed in a systematic manner, to boost optimism, positive affect, and resilience. In healthy participants, PP exercises have consistently increased well-being and decreased depression in studies of over 5000 participants [24]. More recently, trials in patients with coronary heart disease, hypertension, and HIV have found that such programs have had beneficial effects on well-being, depression, and in some cases, health behaviors [25–29].

PP interventions have several potential advantages to other treatment programs, particularly when considering individuals with T2D. First, as opposed to treatments that are applied only to patients with clinical depression or other psychiatric disorders, PP interventions are instead designed to increase positive psychological well-being across a variety of different populations, including those considered to be psychiatrically healthy [30]. This should make the PP intervention more applicable to individuals with T2D who may experience a range of psychiatric symptoms. PP also differs from somewhat-related mindfulness-based stress reduction and self-efficacy interventions [31–33] in that it: (a) specifically targets constructs-positive affect and optimism-linked to superior adherence and outcomes in T2D [3], (b) utilizes validated PP exercises found to be effective across dozens of studies [24], and (c) is simple for patients and does not require the substantial provider training needed for most other interventions.

Despite the potential benefits of an intervention that could promote well-being, reduce distress, and improve self-care in T2D patients, there has been limited study of PP interventions in this patient population. However, results to date have yielded encouraging findings. For example, an online positive affect intervention for T2D patients led to improvements in positive affect and depression, although no significant changes in diabetes-specific efficacy or in health behaviors were found [34]. A related resilience-focused intervention for T2D patients was, however, associated with improvements in HDL cholesterol and fasting blood sugar in a small controlled trial [35], and a one-arm pilot study of a PP intervention also found substantial improvements in psychological outcomes and self-reported health behavior adherence [36].

In the context of these promising but mixed results in T2D patients, there is still a question about whether such PP interventions alone are enough to result in changes in self-care and outcomes, or whether they are better combined with existing behavioral interventions. In the case of the latter scenario, the well-being component would presumably promote motivation, self-efficacy, and optimism, which would allow for greater engagement in the intervention. It is also still unknown how PP interventions compare to other behavioral interventions that have been tested in this population targeting self-efficacy or stress management, which have been shown to affect diabetes-related distress [37,38] and, less consistently, health outcomes [39–42]. Given the clear associations between well-being and outcomes in T2D, along with the promising effects of initial PP-based studies, continued work in this area is needed to ascertain whether well-being interventions can truly impact function, blood sugar, and overall health in this key population.
